# The complete mitochondrial genome of an east African honey bee, *Apis mellifera monticola* Smith (Insecta: Hymenoptera: Apidae)

**DOI:** 10.1080/23802359.2017.1372722

**Published:** 2017-09-01

**Authors:** Amin Eimanifar, Rebecca T. Kimball, Edward L. Braun, Stefan Fuchs, Bernd Grünewald, James D. Ellis

**Affiliations:** a Entomology and Nematology Department, Honey Bee Research and Extension Laboratory, University of Florida, Gainesville, FL, USA;; b Department of Biology, University of Florida, Gainesville, FL, USA;; c Institut für Bienenkunde, Polytechnische Gesellschaft, Goethe-Universität Frankfurt am Main, FB Biowissenschaften, Oberursel, Germany

**Keywords:** *Apis mellifera monticola*, mitogenome, next-generation sequencing, African honey bee

## Abstract

The complete mitochondrial genome of *Apis mellifera monticola* was sequenced and annotated. The genome is 16,343 bp in length and encodes all 37 mitochondrial genes with an A + T content of 84.8%. Gene directions and arrangements are identical to those of other sequenced mitogenomes in *Apis*. Most genes initiated with ATT, though ATG, ATA, and ATC also were used as start codons. All genes terminated with TAA. Four PCG genes, eight tRNAs and both rRNAs are encoded on the heavy strand while all others are coded on the light strand (nine PCGs and 14 tRNAs). Overall, the GC content composed 15.2% of the mitogenome. All of the 22 tRNA genes, ranging from 63 to 78 bp, have a typical cloverleaf structure. A phylogenetic tree showed that *A.m. monticola* clusters with other African subspecies.

*Apis mellifera monticola* Smith (1961) is an African honey bee subspecies, distributed in forests at altitudes of 2000−3000 m in eastern Africa, around Kilimanjaro, Mt. Meru (Tanzania), Elgon, and the highlands of Ethiopia (Sheppard 2015). This subspecies is ecologically isolated and has a disjunct population. It has unique morphometric characters, including longer body hair, dark pigmentation of the abdomen with a narrow yellow stripe on tergite 3, and a very slender abdomen (Drescher 1975). In contrast to *A.m. scutellata*, a related and neighbouring subspecies, it is a very gentle subspecies whose colonies are manageable without requiring protective clothes (Smith 1961; Drescher 1975). Here, we report the complete mitochondrial genome of *A.m. monticola* (GenBank accession no. MF678581) which has not been sequenced previously.

An adult worker honey bee of *A.m. monticola* was obtained from the Ruttner Bee Collection at the Bee Research Institute at Oberursel, Germany (Voucher no. 1626, Kenya, Dietz and Krell, 0°06N, 37°29E). The subspecies identity was confirmed by morphometric evaluation. Total genomic DNA extraction, quantifications, sequencing, mapping, assembling, and annotating were performed following the method published in Eimanifar et al. (2016, 2017). The assembled mitogenome was aligned with those of other *Apis* spp using Mesquite v 3.10 (Maddison and Maddison 2016).

The complete mitogenome sequence of *A.m. monticola* was a classical double-stranded circular molecule which was 16,343 bp in length. The overall nucleotide composition of the major strand of the *A.m. monticola* mitogenome was as follows: A = 43.1%, C = 9.6%, G = 5.6%, and T = 41.6%, with a total A + T content of 84.8%. The mitogenome encoded all 37 genes typically found in animal mitogenomes, including 13 protein-coding genes (PCGs), two ribosomal RNAs, and 22 transfer RNAs. The gene arrangement and structure in the mitogenome of *A.m. monticola* are identical to that of other honey bee subspecies (Eimanifar et al. 2016, 2017). As in other animal mitogenomes, ATP6 and ATP8 share 19 bp, though there were no other overlapping genes or tRNAs. The A + T-rich region of the *A.m. monticola* mitogenome is 827 bp long with 96% A + T.

Twenty two tRNA genes were identified between the rRNA and PCGs, ranging in size from 63 to 78 bp. All tRNAs folded into a conventional cloverleaf shaped secondary structure as identified by tRNAscan-SE (Lowe and Eddy 1997). The sizes of the small ribosomal RNA (12S rRNA) and large ribosomal RNA (16S rRNA) genes were 786 bp and 1371 bp with an A + T content of 81% and 84.7%, respectively.

The total length of all 13 PCGs was 11,048 bp, which accounts for 67.6% of the whole genome sequence. The A + T content of the 13 genes was also high, having 83.3% A + T. Six of the 13 PCGs have an ATT start codon, three have ATA, three have ATG, and one has ATC. All PCGs terminate with TAA. Four PCG genes (ND1, ND4, ND4L ND5), eight tRNAs and both rRNAs were encoded on the heavy strand, while the remaining PCGs and tRNAs were encoded on the light strand.

The phylogenetic relationships of *A.m. monticola* with other honey bees were determined by analysing sequences of 13 PCGs and both rRNAs with maximum likelihood (ML) using RaxML 8.2.10 (Stamatakis 2014) with 1000 bootstrap replicates. *A.m. monticola* clustered with other African subspecies (*A.m. scutellata* and *A.m. capensis*) (Figure 1). The maximum *p*-distance was between *A.m. monticola* and *A. florea* (0.152) and the minimum between *A.m. monticola* and *A.m. scutellata* (0.003).

**Figure 1.**
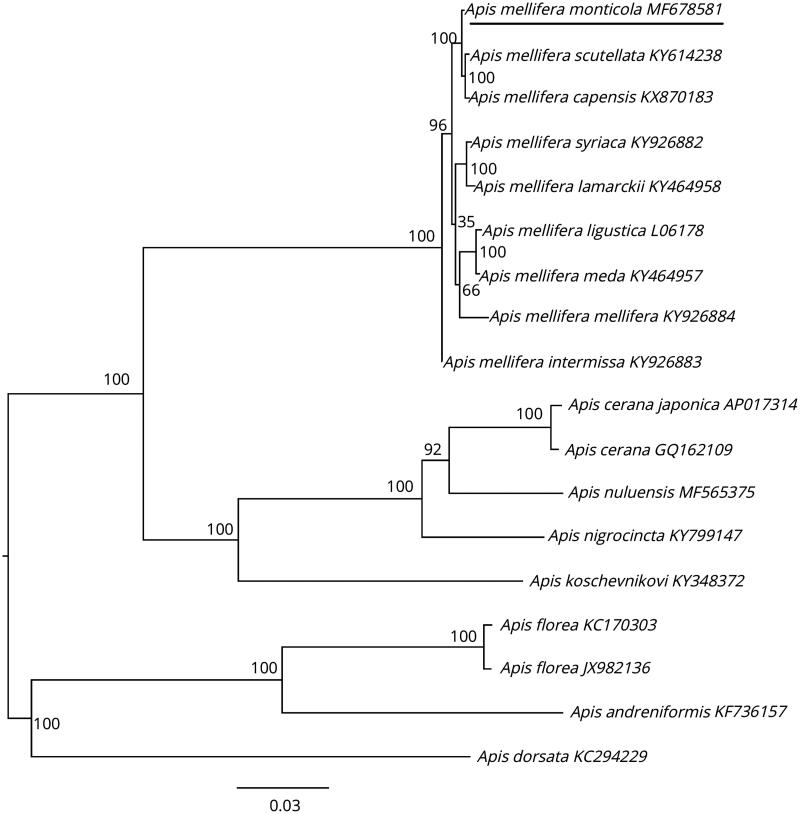
Phylogenetic relationship of *A.m. monticola* based on a concatenated dataset (13 PCGs + two rRNA genes) constructed by maximum likelihood approach. The GTR + G model was applied to each partition. Seventeen mitogenome sequences were obtained from GenBank and included in the tree with their accession numbers. Numbers next to the nodes indicate bootstrap support. The GenBank accession numbers are indicated after the scientific name.
